# Cost-effectiveness of the add-on exenatide to conventional treatment in patients with Parkinson’s disease when considering the coexisting effects of diabetes mellitus

**DOI:** 10.1371/journal.pone.0269006

**Published:** 2022-08-11

**Authors:** Hsuan-Chih Chen, Chen-Yu Wang, Hsiu-Hsi Chen, Horng- Huei Liou

**Affiliations:** 1 Graduate Institute of Clinical Pharmacy, College of Medicine, National Taiwan University, Taipei, Taiwan; 2 School of Pharmacy, College of Medicine, National Taiwan University, Taipei, Taiwan; 3 Department of Pharmacy, National Taiwan University Hospital Yun-Lin Branch, Douliu, Taiwan; 4 National Center for Geriatrics and Welfare Research, National Health Research Institutes, Yunlin, Taiwan; 5 Institute of Epidemiology and Prevention Medicine, College of Public Health, National Taiwan University, Taipei, Taiwan; 6 Department of Neurology, National Taiwan University Hospital, Yunlin Branch, Yunlin, Taiwan; 7 Department of Neurology and Pharmacology, College of Medicine, National Taiwan University, Taipei, Taiwan; Universita degli Studi di Napoli Federico II, ITALY

## Abstract

**Objective:**

This study aims to investigate the cost-effectiveness of the add-on exenatide to conventional pharmacotherapy in patients with Parkinson’s disease (PD) when considering the coexistence of diabetes mellitus (DM).

**Methods:**

We used the Keelung and Community-based Integrated Screening databases to understand the medical utilisation in the Hoehn and Yahr stages of patients with PD. A Markov model with 1-year cycle length and 50-year time horizon was used to assess the cost-effectiveness of add-on exenatide to conventional pharmacotherapy compared to conventional pharmacotherapy alone. All costs were adjusted to the value of the new Taiwanese dollar (NT$) as of the year 2020. One-way sensitivity and probability analyses were performed to test the robustness of the results.

**Results:**

From a societal perspective, the add-on exenatide brought an average of 0.39 quality-adjusted life years (QALYs) gained, and a cost increment of NT$104,744 per person in a 50-year horizon compared to conventional pharmacotherapy. The incremental cost-effectiveness ratio (ICER) was NT$268,333 per QALY gained. As the ICER was less than the gross domestic product per capita (NT$839,558), the add-on exenatide was considered to be very cost-effective in the two models, according to the World Health Organization recommendation. Add-on exenatide had a 96.9% probability of being cost-effective in patients with PD, and a 100% probability of being cost-effective in patients with PD and DM.

**Conclusion:**

Add-on exenatide is cost-effective in PD combined with DM. Considering that DM may be a risk factor for neurodegenerative diseases, exenatide provides both clinical benefits and cost-effectiveness when considering both PD and DM.

## Introduction

Parkinson’s disease (PD) is a progressive neurodegenerative disease that affects 100–200 per 100,000 people over 40 years of age [1]. Population aging is expected to impose an increasing social and economic burden on society [2]. In the United States, the annual medical cost for a patient with PD ranges from US$12,805 to US$23,101 [3,4]. There are geographic differences. In Asia, the mean annual cost per patient is reported as $3,635, which is lower than that in Australia ($7020), Europe ($3,635), and the United States. Nevertheless, the medical cost for PD is almost one-seventh that of the GDP. The cost of illness due to PD is enormous and increases with the disease progression [5–7].

In addition, the total cost for patients with PD escalates with the progression of the Hoehn and Yahr (H-Y) stage [5]. For a patient, slowing disease progression by 10% would have a net monetary benefit of US$29,001 (US$36,362 including income lost), and progression by 20% would have net monetary benefits of US$60,657 (US$75,981 including lost income) [8]. Approaches that slow the progression of PD may greatly reduce expenditure on society.

Thus, PD management mainly focuses on slowing disease progression and providing pharmacotherapy for symptomatic control. However, no currently available drugs can inhibit disease progression [9]. Exenatide, a glucagon-like peptide-1(GLP-1), is a second-line treatment for type 2 diabetes mellitus (DM) and has demonstrated clinical benefits for patients with PD in randomised controlled trials (RCT) in 2013 [10,11]. When exenatide is added to conventional pharmacotherapy for PD, it delays disease progression [10], but its cost is relatively high.

Furthermore, the relationship between PD and DM has been demonstrated in both epidemiological and molecular biology studies, which show that preceding type 2 DM increases the incidence and progression of PD [12–23]. As a result, the combined use of exenatide with standard PD treatment may have different roles in the population with both PD and type 2 DM compared to the population with only PD. With regard to dual diseases that modify exenatide, cost-effectiveness must be established.

Considering the significant clinical benefits for patients with PD, but the relatively high cost of exenatide, and the relationship between PD and DM, we conducted cost-effectiveness analyses on the add-on exenatide to the conventional treatment in patients with PD when considering the coexisting effect of DM.

## Materials and methods

The Markov decision model was used to analyse the cost-effectiveness of add-on exenatide for PD because PD is a lifetime disease and needs chronic pharmacological treatment. Our Markov model was a modified version of those used in previous studies with a cycle length of a year to capture cost-effectiveness [24,25]. The model for assessing cost effectiveness was constructed to represent the real-world situation. The input parameters for the simulation were obtained from the Keelung and community-based integrated screening database (KCIS) [26], the National Health Insurance Research Database (NHIRD) [27], and currently available evidence. The details of PD screening are described elsewhere [28,29]. We obtained information about the characteristics and medical utilisation of patients with PD from the KCIS database and created an appropriate model for subsequent cost-effectiveness analysis [28,29].

### Framing the model

#### Target population

A cost-effectiveness analysis was conducted with a hypothetical community-based cohort of 1,000 people with health status or DM according to the prevalence.

#### Study perspective

In this study, we used the societal perspective cost-effectiveness analysis.

#### Comparators

This study includes two interventions. One is conventional pharmacotherapy and the other is conventional pharmacotherapy with add-on exenatide. The former includes conventional therapy for both PD and DM.

The gold standard for treating PD is levodopa and can be combined with dopamine agonists, monoamine oxidase (MAO)-B inhibitors, amantadine, catechol-O-methyl transferase (COMT) inhibitors, and anticholinergic agents, depending on the severity of symptoms. The conventional therapy for DM consists of metformin monotherapy, sulfonylurea monotherapy, combination use of metformin and sulfonylurea, and combination use of metformin and thiazolidinedione (TZD). Metformin is the first-line therapy for patients with type 2 DM. Patients start metformin immediately after they fail to achieve the glycaemic target by lifestyle modification or they may start metformin therapy immediately if they have relatively high and uncontrolled HbA1c levels [30]. As the disease progresses, second-line drugs that could be added to metformin include sulfonylurea, TZD, dipeptidyl peptidase (DPP)-4 inhibitors, sodium-glucose co-transporter-2 (SGLT-2) inhibitors, GLP-1 agonists, and insulin [30].

#### Time horizon

A 50-year time horizon was used to assess the cost-effectiveness of the add-on exenatide to conventional pharmacotherapy compared to conventional pharmacotherapy alone.

#### Discount rate

The discounting rate was set at 3% with a range of 0%–6%, based on the cost-effectiveness analysis guidelines of the National Institute of Health Technology Assessment (HTA) of Taiwan [31].

### Outcome of the model

The outcomes of the models were life expectancy, quality-adjusted life years (QALYs), and costs. By comparing the two interventions, the incremental cost-effectiveness can be calculated for conventional therapy versus add-on exenatide.

### Structuring the model

The Markov decision model was used to analyse the cost-effectiveness of add-on exenatide for PD because PD is a lifetime disease and needs chronic pharmacological treatment. The Markov model used by us is a modified version of that used in previous studies with a cycle length of one year to capture cost-effectiveness [24,25].

We assumed that once the patients entered the exenatide group, they continued using exenatide until the end of the study or until death. The symbol 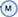 at the end of each treatment arm indicates a Markov chain for the process of PD evolving with time, and the two strategies had the same evolving condition.

In the Markov model, data regarding the initial state of the H-Y stage were based on the prevalence in different stages derived from the results from the KCIS databases.

We hypothesised that the progression of PD is irreversible owing to its natural history. This assumption has been adopted in many previous studies [24,25,32–34].

We considered the preceding type 2 DM and PD simultaneously. Because of the distinct natural history of these two diseases, we postulated that DM would precede PD in this model according to the age-specific incidence rate and real-world situations [29,35].

A cost-effectiveness analysis was conducted with a hypothetical community-based cohort of 1,000 people with health status or DM according to their prevalence. The participants entered the cohort at the age of 40 years, and the time horizon of the study was 50 years (i.e. 50 cycles). We defined healthy people as the beginning of the model to represent the progression from health to disease, in which PD incidence is affected by DM, as mentioned earlier.

#### Model states

**Fig 1:**
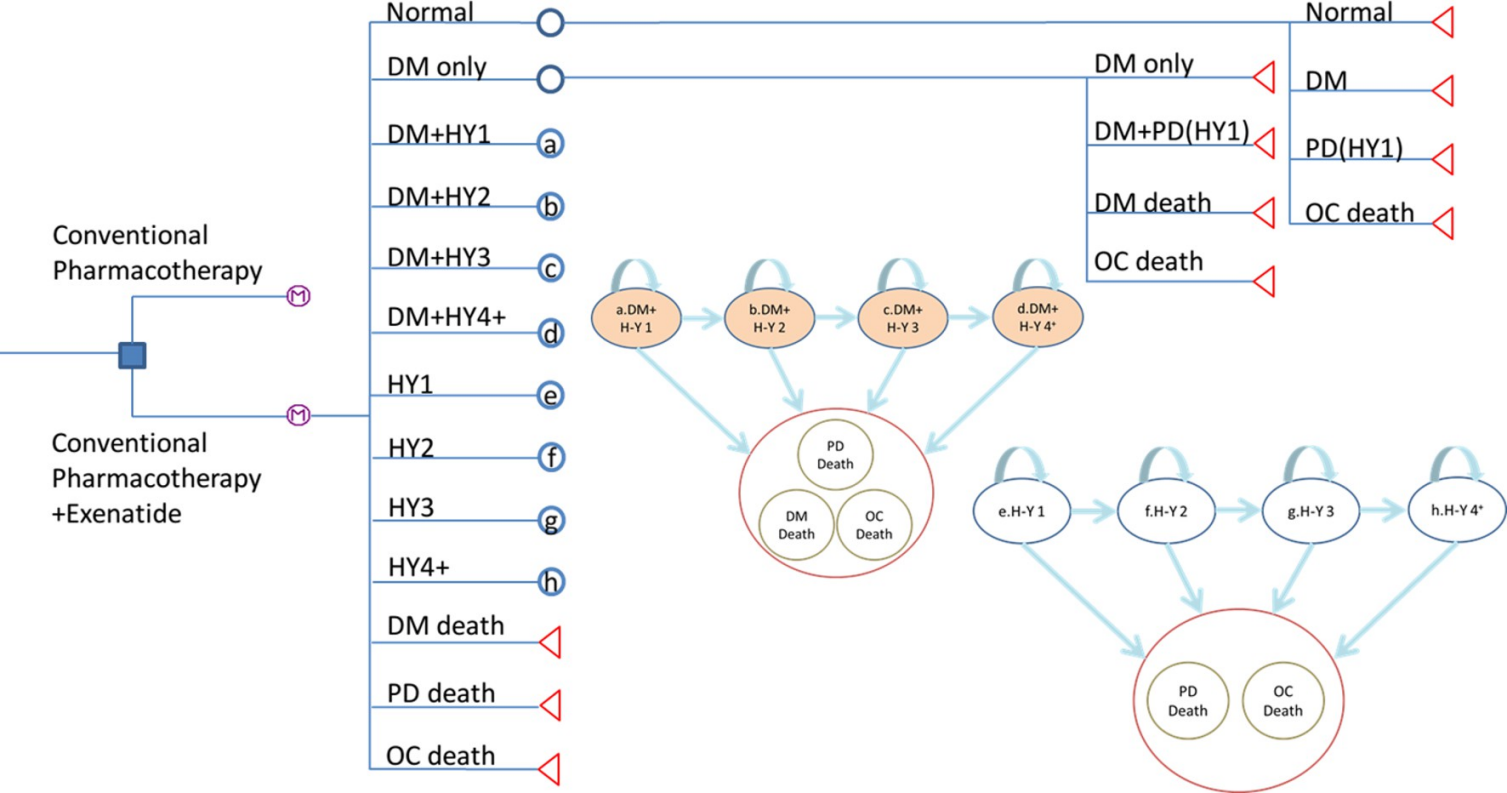
The Markov model for cost-effectiveness analysis for the add-on exenatide effect on PD with DM.

There are thirteen different states in the tree-based Markov model as shown in **Fig 1**. The alphabet in the nodes behind each state represents the corresponding state of Markov model they would enter. In the model, people started from “normal” state. They would develop to DM, PD or stay in normal in each cycle. H-Y 1 was the initial stage of incident PD patients. Considering the fact that onset age of DM was younger than PD, the comorbidity of DM and PD occurred after DM. The irreversible features of PD in terms of H-Y stage were remained in this model. The background age-specific death rate was considered identical for all state.

#### Likelihood of events

The probabilities of transitions between states were retrieved from the KCIS database or literature. The upper and lower limits were simply adapted as indicated in the literature. The 95% CI was used as the upper and lower limit for the sensitivity analysis.

#### Probability of developing disease

The probabilities of developing PD or DM were retrieved from Taiwanese studies [27,35]. The average and 95% CI were obtained from multiple years of data. The 50-year time horizon of the hypothetical cohort was further divided into 40–49, 50–59, 60–69, 70–79, and > 80 years. The elevated probability of developing PD in preceding DM came from the probability of developing PD multiplied by the age-specific ratio from studies in Taiwan [23,36]. In the age group 40–60, the ratio was 2.20, and 1.5 for above 60 years old.

#### H-Y stage transition probabilities

**Fig 2:**
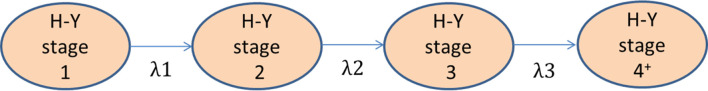
Model for estimating H-Y stage transition probabilities.

There are four conditions in this section: (1) PD under conventional pharmacotherapy, (2) PD under conventional pharmacotherapy and exenatide, (3) PD comorbid with DM under conventional pharmacotherapy, and (4) PD comorbid with DM under conventional pharmacotherapy and exenatide. **Table 1** shows the annual transition rates among the H-Y stages (**Fig 2)** from which the annual transition probability can be derived using the Markov process model [28]. Note that the slow transition from H-Y 2 to H-Y-3 (**λ2**) resulted from the slow progression for patients in H-Y 2 stage following the estimated results from Liou et al [28] that patients spent an average of 6.6 years in H-Y 2 stage, which was longer than 2.8 years in H-Y 1 and 1.4 years in H-Y 3.

**Table 1:** H-Y stage transition rate in different condition.

	λ1	λ2	λ3
**PD** *	0.3237	0.068	0.3192
**PD with Exenatide**	0.3065 (= 0.3237*0.947)	0.0163 (= 0.068*0.239)	0.0658 (= 0.3192*0.206)
**PD+DM** *	0.4369 (= 0.3237*1.35)	0.1462 (= 0.068*2.15)	0.3192
**PD+DM with Exenatide**	0.4137 (= 0.4369*0.947)	0.0349 (= 0.1462*0.239)	0.0210 (= 0.3192*0.206)

* Conventional pharmacotherapy.

#### Probability of death

(1) Death in PD

The probabilities of death in PD were retrieved from previous studies in Taiwan that used the KCIS and Ilan database [37]. In contrast to other probabilities of death, the deaths in PD were H-Y stage-specific instead of being age-dependent. We did not consider the effects of exenatide on the death of PD due to the lack of long-term trials and to avoid overestimation.

(2) Death in DM

Age-specific probabilities of death in DM were obtained from the study of death in patients with DM in Taiwan with National Health Insurance Research Database (NHIRD) [38]. The 50 years were further divided into 40–49, 50–59, 60–69, 70–74, and > 75 years.

Regarding exenatide, the incidence of DM complications has significantly declined in many randomised controlled trials, but we could not measure exact reduction in mortality because we did not have an exact value for the reduction in complication [39–42]. Consequently, we referred to the projected 10 and 20 life years (LY) gained in the study by Minshall et al. and estimated the reduction in mortality by life table [43,44]. We obtained an estimated efficacy of 0.88 (95% CI 0.86–0.90) in the add-on exenatide group compared to conventional therapy. By directly multiplying age-specific DM death by 0.88, we obtained the probabilities of DM death with add-on exenatide.

(3) Death due to other causes

Age-specific deaths without DM as a cause in Taiwanese people were obtained from Taiwanese government statistics [45]. The time horizon was divided into 40–49, 50–59, 60–69, 70–79, and > 80 years. The death rates of these five timeframes were represented by the probability of death at the ages of 45, 55, 65, 75, and 80 years, respectively. The upper and lower limits were based on the upper and lower ranges of each timeframe.

#### Cost

We used the societal perspective cost-effectiveness analysis. Hence, costs, including direct medical costs and indirect costs, were considered. The former includes outpatient clinics, inpatients, examinations, laboratory tests, and drugs. Indirect costs include the loss of productivity. Costs were estimated from the data available from KCIS, Taiwanese literature, and government. All costs were presented in New Taiwanese Dollars (NT$) and adjusted to the value in 2020 according to the medical component of the consumer price index (CPI) in Taiwan [46].

(1) Costs of PD

The medical costs of PD were obtained from the KCIS and categorised according to the H-Y stage. The cost of H-Y 1 in PD comorbid with DM could not be determined due to the relatively small sample size in our cohort; therefore, there were no patients with PD and DM in H-Y 1. We substituted the costs of H-Y 1 in patients with PD only for these missing data because there would be a small difference in cost between early PD and early PD with DM. We also included the costs of home care in H-Y stages 3 and 4^+^ as part of the direct cost [47]. Regarding indirect costs, we multiplied the average income per person by the productivity loss derived from previous literature. The average income was retrieved from the Survey of Family Income and Expenditure in 2015, and income of 65 years and above was used on account of the characteristics of older age in PD [48].

(2) Cost of DM

We considered the medical costs between 2000 and 2009 in the NHIRD owing to the scarcity of longitudinal trials, especially for comparing the costs of conventional treatment and treatment with exenatide in Taiwan [49]. The costs incurred between 2000 and 2009 were excluded from exenatide because they had not been approved until 2010. We conservatively estimated that the difference between the two strategies was productivity lost by different mortality rates due to insufficient data on the reduction in total medical costs by exenatide. We reckoned age-specific costs by the average costs and reported linear trends in costs of DM in Taiwan, which increased by about NT$15,000 per capita every 10 years [50].

All the input parameters with their plausible ranges and corresponding references are listed in **Table 2.** The costs of outpatient clinics and hospitalisation, and the total costs of PD and PD combined with DM are presented in **Table 3.**

**Table 2:** Parameter input and data sources for the cost-effectiveness analysis.

Parameters	Input value	Range for Sensitivity analysis	Source(s)
**Prevalence of DM**			**[35]**
40–49 years old	0.0605	-	
**Incidences (by age group)**			
**PD**			**[51]**
40–49 years old	0.0	-	
50–59 years old	0.000211	0.000193–0.000229	
60–69 years old	0.000995	0.000773–0.001218	
70–79 years old	0.003005	0.002577–0.003435	
≥80 years old	0.003699	0.003280–0.004118	
**DM**			**[35]**
40–49 years old	0.00964	0.00911–0.01017	
50–59 years old	0.00964	0.00911–0.01017	
60–69 years old	0.01906	0.01699–0.02112	
70–79 years old	0.01906	0.01699–0.02112	
≥80 years old	0.01637	0.01419–0.01854	
**PD comorbid with DM**			**[23,36,51]**
40–49 years old	0.0	-	
50–59 years old	0.000464	0.000371–0.000578	
60–69 years old	0.001542	0.001453–0.001711	
70–79 years old	0.004658	0.004387–0.005169	
≥80 years old	0.005733	0.005401–0.006362	
**H-Y stage transition rate** **PD with conventional therapy**			**[37,52–54]**
H-Y 1 –> H-Y 2	0.3237	-	
H-Y 2 –> H-Y 3	0.068	-	
H-Y 3 –> H-Y 4^+^	0.3192	-	
**PD with conventional therapy and exenatide**			**[10,11,37,52–54]**
H-Y 1 –> H-Y 2	0.3065	-	
H-Y 2 –> H-Y 3	0.0163	-	
H-Y 3 –> H-Y 4^+^	0.0658	-	
**PD comorbid with DM**			**[27,52–56]**
H-Y 1 –> H-Y 2	0.4369	-	
H-Y 2 –> H-Y 3	0.1462	-	
H-Y 3 –> H-Y 4^+^	0.3192	-	
**PD comorbid with DM with conventional therapy and exenatide**			**[10–12,28,33,52–54]**
H-Y 1 –> H-Y 2	0.4137	-	
H-Y 2 –> H-Y 3	0.0349	-	
H-Y 3 –> H-Y 4^+^	0.0658	-	
**Mortality**			**[38]**
**DM (by age group)**			
40–49 years old	0.01510	0.0099–0.0203	
50–59 years old	0.02195	0.0123–0.0350	
60–69 years old	0.03931	0.0273–0.0547	
70–74 years old	0.05288	0.0486–0.0580	
≥75 years old	0.08150	0.0778–0.0863	
**DM with exenatide (by age group)**			**[38,57,58]**
40–49 years old	0.01329	0.0129–0.0136	
50–59 years old	0.01932	0.0189–0.0198	
60–69 years old	0.03459	0.0338–0.0353	
70–74 years old	0.04653	0.0455–0.0476	
≥75 years old	0.07172	0.0701–0.0734	
**Other causes (by age group)**			**[59]**
40–49 years old	0.00266	0.0017–0.0035	
50–59 years old	0.00507	0.0038–0.0065	
60–69 years old	0.01028	0.0070–0.0148	
70–79 years old	0.02677	0.0164–0.0393	
≥80 years old	0.05722	0.0432–0.0628	
**PD (by HY stage)**			**[37,52]**
H-Y 1	0.01	-	
H-Y 2	0.048	-	
H-Y 3	0.080	-	
H-Y 4+	0.199	-	
**Effects of exenatide**			
DM mortality	0.88	0.86–0.90	**[43,44]**
H-Y transition rate 1->2	0.981	0.95–0.99	**[10,11]**
H-Y transition rate 2->3	0.245	0.20–0.32	**[10,11]**
H-Y transition rate 3->4^+^	0.214	0.17–0.28	**[10,11]**
**Costs**			
**PD medical costs**			**NHIRD**
		
H-Y 1	NT$21821.9*	-	
H-Y 2	NT$69074.2	17470.5–254268.4	
H-Y 3	NT$71969.3	22218.5–152150.8	
H-Y 4^+^	NT$133558.1	50890.6–172994.6	
**PD comorbid with DM Medical costs**			
			**NHIRD**
H-Y 1	NT$21821.9	-	
H-Y 2	NT$90217.1	24685.3–264341	
H-Y 3	NT$77079.1	43927.4–123334.5	
H-Y 4^+^	NT$148740.9	-	
**Home care of PD**			
H-Y 1	NT$0	-	
H-Y 2	NT$0	-	
H-Y 3	NT$22220	-	
H-Y 4^+^	NT$22220	-	
**Non-medical costs** **Productivity lost**			**[48,60]**
H-Y 1	NT$17261.2	5218.5–29304.0	
H-Y 2	NT$52185.1	40142.4–64227.8	
H-Y 3	NT$95538.9	83496.2–107581.6	
H-Y 4^+^	NT$54593.7	42550.9–66636.4	
**DM medical costs** **(by age group)**			**[49,50]**
40–49 years old	NT$43903.6	39887.8–47919.4	
50–59 years old	NT$58903.6	54887.8–62919.4	
60–69 years old	NT$73903.6	69887.8–77919.4	
70–79 years old	NT$88903.6	84887.8–92919.4	
≥80 years old	NT$103903.6	99887.8–107919.4	
**DM non-medical costs** **Productivity lost** **(by age group)**			**[48]**
40–49 years old	NT$635049	-	
50–59 years old	NT$605055.6	-	
60–69 years old	NT$ 481893	-	
**Exenatide**	NT$33926.8	-	
**Utilities**			
**PD**			**[56]**
H-Y 1	0.708	0.638–0.778	
H-Y 2	0.678	0.608–0.748	
H-Y 3	0.622	0.552–0.692	
H-Y 4^+^	0.499	0.429–0.569	
**DM**			**[61]**
Difference with non-DM	-0.04	0.0352–0.0448	
Annual decline	-0.003	0.00214–0.00387	
**Normal (by age group)**			**[62]**
40–64 years old	0.92	0.74–1.00	
≥65 years old	0.84	0.39–1.00	
**DM with exenatide**	+0.08	0.06–0.10	**[63,64]**
**Discount rate**	0.0300	0.0000–0.0500	**[31]**

**Table 3:** Medical cost of PD only and PD combined with DM per patient during one year.

	PD only (N = 45)				PD combined with DM (N = 37)			
	H-Y 1	H-Y 2	H-Y 3	H-Y 4^+^	H-Y 1	H-Y 2	H-Y 3	H-Y 4^+^
**No. of patients**	0	29	14	2	1	28	7	1
Age	-	71.3	73.75	76.8	65.1	70.1	73.4	63.5
Medical costs (NT$)								
Outpatient costs	-							
Mean	-	36,787.4	43,056.2	90,327.2	21,821.9	50,240.5	41,407.6	24,708.0
Range	-	100,476.8	74,392.9	78,873.1	0	29,435.0	47,566.7	0
5th percentile	-	9,060.1	16,047.1	50,890.6	21,821.9	21,176.1	21,185.1	24,708.0
25th percentile	-	19,942.7	24,741.9	50,890.6	21,821.9	27,305.0	33,845.5	24,708.0
Median	-	33,416.0	32,152.9	90,327.2	21,821.9	40,146.1	41,407.6	24,708.0
75th percentile	-	45,136.4	62,812.1	129,763.7	21,821.9	51,820.4	60,143.5	24,708.0
95th percentile	-	103,338.2	90,439.9	129,763.7	21,821.9	97,891.5	68,751.8	24,708.0
Inpatient costs	-							
Mean	-	32,286.8	28,913.1	43,230.9	0	39,976.6	35,671.5	124,032.9
Range	-	142,519.8	55,539.5	0.0	0	162,940.3	31,840.3	0.0
5th percentile	-	8,410.4	6,171.4	43,230.9	0	3,509.2	22,742.3	124,032.9
25th percentile	-	14,975.4	16,956.4	43,230.9	0	19,389.6	22,742.3	124,032.9
Median	-	19,022.5	24,186.4	43,230.9	0	27,814.4	29,689.5	124,032.9
75th percentile	-	35,186.9	40,267.4	43,230.9	0	52,665.4	54,582.7	124,032.9
95th percentile	-	150,930.2	61,710.9	43,230.9	0	166,449.5	54,582.7	124,032.9
**Total**	-	69,074.2	71,969.3	133,558.1	21,821.9	90,217.1	77,079.1	148,740.9
**Range**	-	17,470.5–254,268.4	22,218.5–152,150.8	50,890.6–172,994.6		24,685.3–264,341	43,927.4–123,334.5	0

#### Utilities

All utility values were obtained from the literature. The utilities of different H-Y stages were obtained from a Japanese study, and the utilities of DM and its annual declines were retrieved from the SHIELD longitudinal study in the United Kingdom [56,61]. For patients with both diseases, utilities were calculated by subtracting utilities with DM from those in each H-Y stage based on the assumption that PD and DM were independent of health utilities. The utilities of people stay in normal status were obtained from the Chinese literature [62]. Utility changes by exenatide are estimated by two studies, in which exenatide increases utility by 0.08.

### Analysing the model

The base case analysis in this study was conducted from a societal perspective with a cohort of 1,000 patients.

The initial distribution of the patients was based on the prevalence of DM at 40 years of age, and the other was in a normal state. The prevalence of PD was almost zero at the initial age; therefore, we could ignore it. The same distribution was used for both the interventions in this cohort.

The main results were presented as incremental cost-effectiveness ratios (ICERs), including incremental cost per life year (LY) gained and incremental cost per QALY gained.

Unlike in the United Kingdom and the United States, there was no official threshold for the willingness-to-pay (WTP) for a QALY gained in Taiwan. Therefore, the WHO recommendations were applied in our study to check the cost-effectiveness of our results [65]. If the ICER is less than the GDP per capita than the intervention, it is believed to be ‘very cost-effective’, and if the ICER is between 1 and 3 times the GDP per capita than the intervention, it is considered to be ‘cost-effectiveness’. In 2020, Taiwan’s GDP per capita was NT$839,558 [66].

#### Sensitivity analysis

One-way sensitivity analyses of all input parameters, including probabilities, costs, proportion of effects of exenatide, utilities, and discounting rate, were performed with the upper and lower limits of each parameter. One-way sensitivity analysis was used to determine whether the uncertainty of the parameters would affect the results of the cost-effectiveness analysis. The results were presented with a tornado plot, where the most influential parameter was lined on the top of the plot, followed by the rest of the parameters, according to the scale of impact on the results.

We performed probabilistic sensitivity analysis (PSA) to test the robustness of the analysis. In the PSA, 1,000 Monte Carlo simulations were performed. The values of each parameter were randomly drawn from the plausible ranges in each simulation. The estimated ICERs were calculated for each simulation, and the simulation results were presented using an ICER plane. A cost-effectiveness acceptability curve (CEAC) was also presented to show the relationship between the WTP threshold and the probability of being cost-effective in each intervention group. Additionally, several scenario sensitivity analyses were conducted to examine the results in special situations.

The situation includes: (1) exenatide reduces PD incidence by 20% in patients with DM, and (2) the effect of exenatide can be applied to the early stages of PD, so we replaced the H-Y transition rates of 1->2 with that of 2->3.

#### Statistical software

The analyses were performed using SAS9.4 (SAS Institute, Cary NC), Microsoft ^®^ Excel (Microsoft Corp., Redmond, WA), and TreeAge Pro (TreeAge Software Inc.).

### Ethics statement

Our study does not involve individual data. Parameters pertaining to our decision tree were derived from literature. There is no requirement for an IRB approval.

## Results

### Base-case analysis

From the social perspective (**Table 4**), the add-on exenatide brought about an average of 0.39 QALYs gained and a cost increment of NT$117,890 per person in a 50-year horizon compared to the conventional pharmacotherapy. The ICER was NT$302,011 per QALY gained. The ICER per QALY gained was less than the GDP per capita of Taiwan in 2020 (NT$839,558), and add-on exenatide was considered to be highly cost-effective according to the WHO recommendation [67]. With the same setting, the add-on exenatide resulted in saving of 0.14 LYs with an ICER per LY saved of NT$818,111.

**Table 4:** Results of the cost-effectiveness analyses: Base-case analysis and scenario sensitivity analyses in Aim 3 (PD + DM).

	Average LYs per person		Average QALYs per person		Average costs per person		ICER: Cost per LY saved	ICER Cost per QALY saved
	Total	Incremental	Total	Incremental	Total	Incremental		
Base-case analysis								
Conventional	21.75		18.45		1529090			
Conventional+ exenatide	21.90	0.14	18.84	0.39	1633835	104744	726881	268333
Scenario sensitivity analysis: exenatide reduce PD incidence by 30%								
Conventional	21.75		18.45		1529091			
Conventional+ exenatide	21.90	0.15	18.84	0.39	1633802	104711	719598	266797
Scenario sensitivity analysis: exenatide has effects on early-stage patient with PD								
Conventional	21.75		18.45		1528886			
Conventional+ exenatide	21.91	0.15	18.84	0.40	1629200	100314	653375	253616

### One-way sensitivity analysis and probabilistic sensitivity analysis

**Fig 3:**
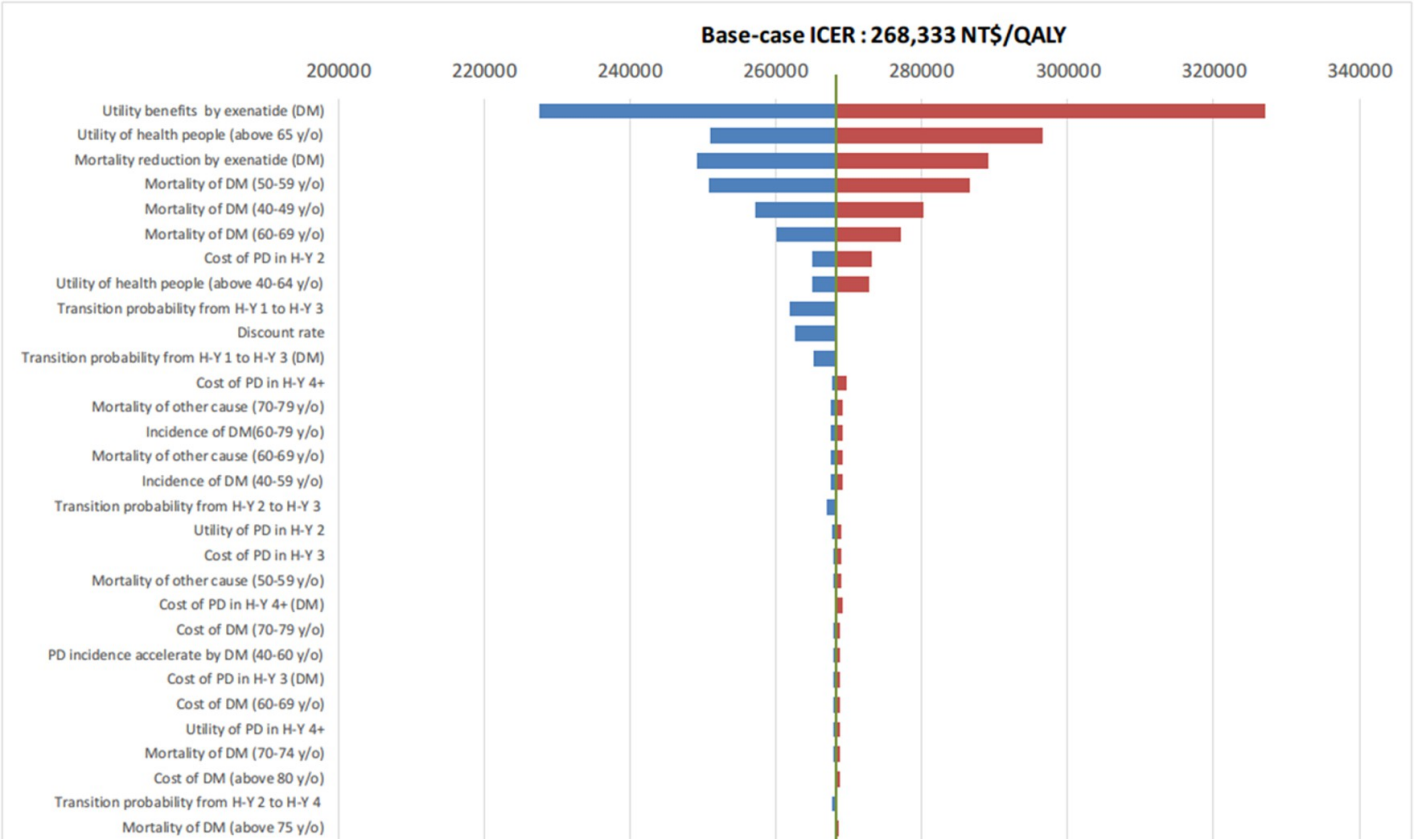
Tornado plot of the one-way sensitivity analysis of all input parameters of the cost-effectiveness analysis comparing conventional pharmacotherapy + exenatide with conventional pharmacotherapy in PD with DM.

**Fig 4:**
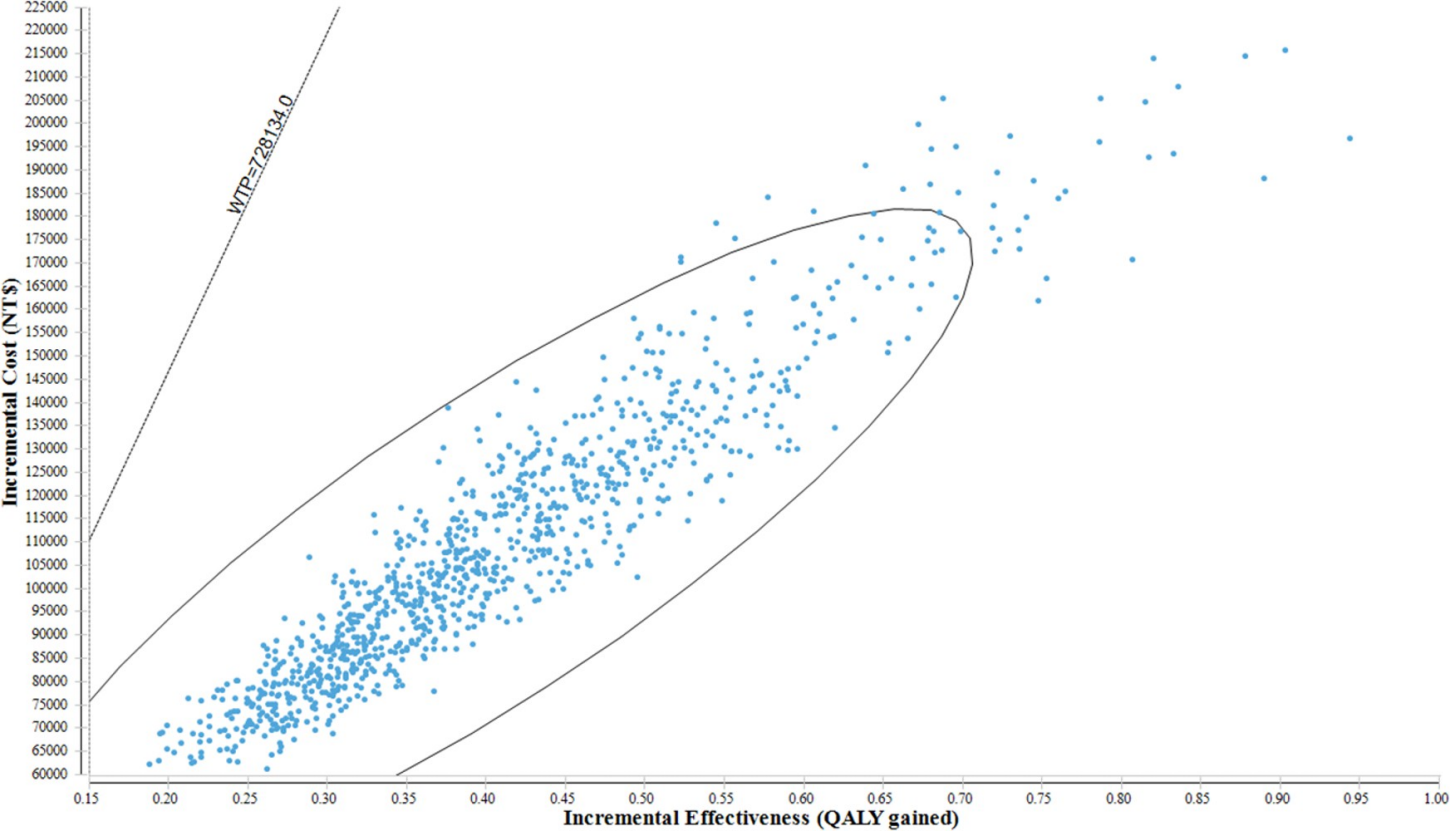
Incremental cost-effectiveness ratio (ICER) scatter plot of the conventional pharmacotherapy + exenatide vs. conventional pharmacotherapy in PD with DM.

**Fig 5:**
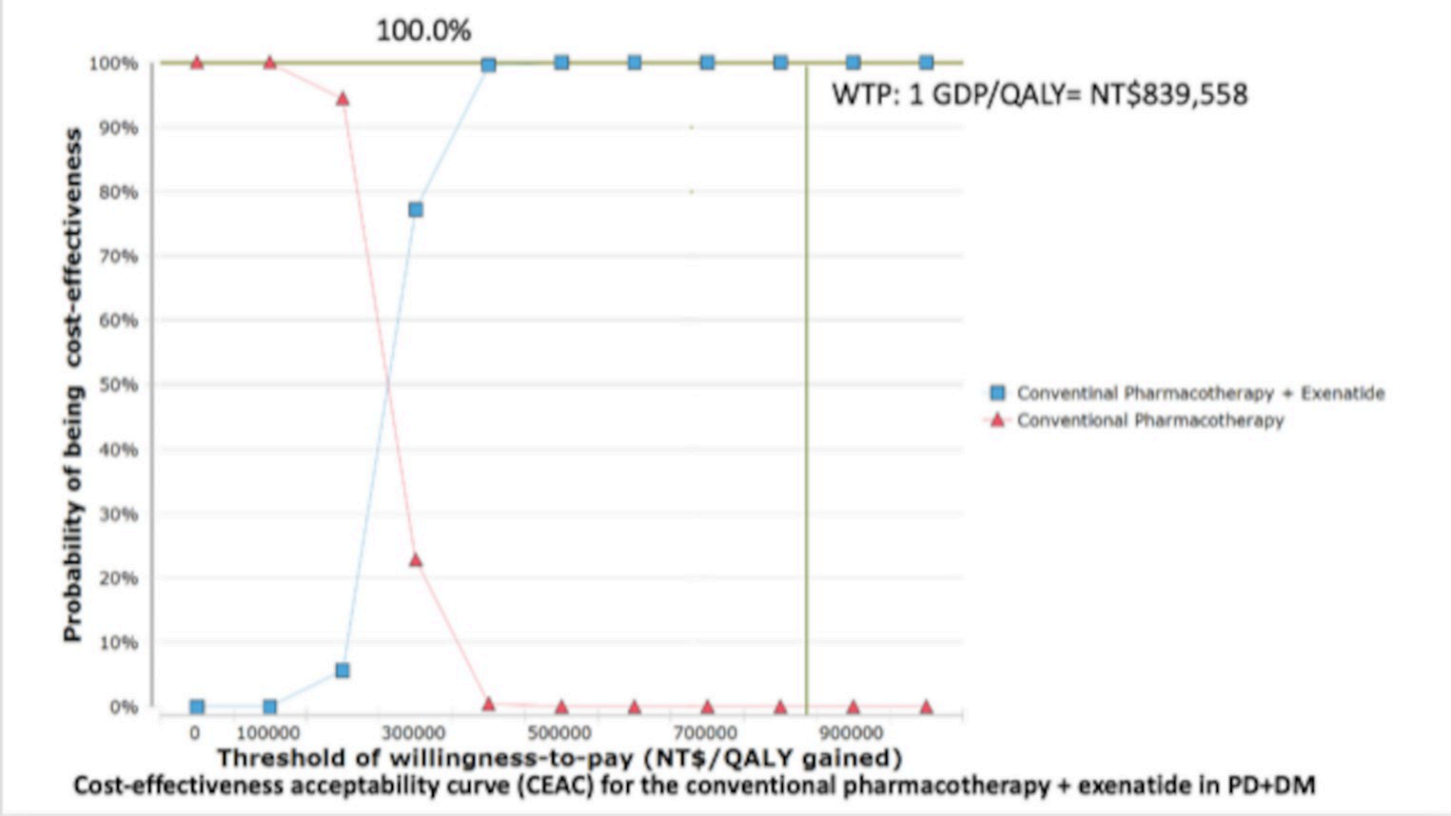
Cost-effectiveness acceptability curve (CEAC) for the conventional pharmacotherapy + exenatide in PD with DM.

**Fig 3** shows a tornado plot of one-way sensitivity for Aim 3. The details of the tornado are described above. The utilities ranked first and second in this analysis because the incidence of PD was relatively small in the population, and the simulative cohort spent most of the time in the normal or DM groups in our model. Therefore, the utility of DM or normal drastically affected the results. However, if GDP per capita is used as the threshold of WTP, none of the values of the input parameters have deviated the results of cost-effectiveness. In the probability sensitivity analyses (**Figs 4** and **5**), the add-on exenatide had a 100% probability of being very cost-effective with a WTP for GDP per capita.

### Scenario sensitivity analyses

The analysis with early-stage effects result in 0.40 QALYs gained and a cost increment of NT$112,904 per person with an ICER of 285,447 per QALY gained, and LYs gained per person with an ICER of 735,379 per LY saved.

## Discussion

Our study found that if the government spends an extra cost of NT$117,890 per person for patients with DM in a 50-year horizon, then an average of 0.39 QALYs per person could be gained. The results suggested that add-on exenatide was more cost-effective than conventional pharmacotherapy.

### Relationship between DM and PD

The relationship between DM and PD has been studied for several years. There is growing evidence that preceding type 2 DM increases the risk of PD and accelerates its progression [12–16,18–23]. In our model, we hypothesised that individuals may progress to DM first and then to PD. This is confirmed because the age-specific incidences are different between the two diseases in which people are vulnerable to DM since 40 years of age, whereas those above 60 years of age are vulnerable to DM in PD. Moreover, molecular-based studies have indicated that the pathophysiology of DM may contribute to PD through several pathways, in which PI3K/AKT may play a key role [68,69]. Substantial evidence suggests that the loss of AKT signalling is involved in type 2 DM and PD.

Insulin not only regulates glucose homeostasis but also acts as an important homeostatic factor in the brain [70]. It activates its downstream PI3K/AKT pathway, which regulates a variety of important functions that are typically disrupted in PD, including apoptosis, autophagy, inflammation, nerve cell metabolism, protein synthesis, and synaptic plasticity [71]. Studies have shown marked loss of insulin receptor mRNA in the substantia nigra pars compacta (SNpc) of patients with PD and increased insulin resistance compared to age-matched controls [72,73].

AKT acts as a master regulator of cellular function [74]. Results from experimental models indicate that inhibition of AKT signalling leads to dopaminergic cell death, and dysregulation of AKT signalling may affect the expression of alpha-synuclein in PD [75,76].

Although insulin resistance is insufficient evidence that DM increases the risk of PD, it remains to be seen whether brain insulin resistance is due to impaired transit of insulin through the blood-brain barrier or if the neurons themselves become directly insensitive to the actions of insulin, a combination, or both. Taken together, the DM dose has a negative impact on PD.

In our model, the transition probabilities of the H-Y stage are also accelerated by DM, but there is no published literature on how DM severity (such as HbA1c) contributes to PD progression, so we can only consider the condition with or without DM.

### Exenatide and conventional pharmacotherapy in DM

As mentioned above, neuroprotection by a GLP-1 agonist comes from the activation of the AKT pathway, which also benefits other insulin-resistant cells in DM. There might be synergic effects of exenatide when treating patients with PD and DM, but we conservatively assumed that ameliorating the progression of PD is the same in PD only and PD comorbid with DM.

In our model, conventional therapy in patients with DM includes metformin, sulfonylurea, thiazolidinedione, and their combination. Several studies have hypothesised that drug use might affect the risk of PD, but so far, there has been an obvious correlation between these drugs and PD. Metformin has been studied for its versatile ability to modify diseases, and its anti-inflammatory and antioxidant properties may be advantageous for PD [68]. However, experimental models have revealed mixed effects on PD. Epidemiological data also suggest neutral outcomes in the risk of PD incidence [22].

A Taiwanese study showed that sulfonylurea might have detrimental effects on the incidence of PD, but the underlying mechanism is uncertain [22]. Moreover, the detrimental effects disappeared in combination with metformin [22]. Some have suggested that the side effects of hypoglycaemia by sulfonylurea are one of the reasons, but more evidence is needed to elucidate the relationship between sulfonylurea and PD.

Taken together, it seems reasonable to assume that the DM drugs used in conventional pharmacotherapy are independent of PD progression. As for the interaction between DM and PD drugs, we also considered their independence due to the lack of published evidence.

### Cost of DM

We retrieved the costs directly from the Taiwanese literature, which analyses the total cost of patients with DM with the NHIRD from 2000 to 2009 [49]. The pharmacotherapy of DM included the drugs mentioned above during this period except exenatide which had not been approved until 2010. We assigned the same DM costs in the two strategies because we could not obtain a direct reduction in exenatide intervention. Studies have suggested that DM-related costs are reduced owing to fewer complications [57,63,64]. To avoid model complexity, we did not consider complications, which made the estimation of cost diminishing in DM with exenatide unattainable. We still have cost-effective outcomes even with underestimation; therefore, more cost-effective results could be expected in the future.

### Scenario analyses of effects on early-stage and prevention

In scenario analyses, early intervention with exenatide was more cost-effective than base-case analysis. Based on the mechanism of neuroprotection, it is reasonable to assume that PD is prevented. If future work proves that the scenario is true, the cost-effectiveness of exenatide will be enormous.

Overall, we demonstrated the cost-effectiveness of exenatide in a population-based model of PD combined with DM. As growing evidence considers the negative impacts of DM on other neurodegenerative diseases such as AD, treatments that can provide neuroprotection and reverse the deterioration of neuronal cells will be the first choice for those who are genetically or environmentally prone to the development of degenerative nerve diseases [68,77–80]. Exenatide, which can provide clinical benefits and reduce the economic burden, is potentially another multiple disease-modifying drug.

Our study has several strengths. First, several local data are applied to demonstrate cost-effectiveness. Compared to applying data from studies in other countries, the local data were considered to be a more suitable and reliable source for representing the true effect of add-on exenatide in Taiwan. There are many differences in transition probabilities in H-Y stages due to the distinct characteristics of patients and treatment [28,33,53,54,81]. The local data in our model truly reflect the situation of conventional pharmacotherapy treatment in Taiwan. Other input parameters were derived from the NHIRD and studies in Taiwan if applicable. Modifications to the input parameters obtained from Western countries were also made to account for racial disparities. Second, our model is the first to evaluate the cost-effectiveness of the add-on exenatide where DM was considered in the meantime. Such a model design not only reflects the clinical situation in which type 2 DM precedes PD, but also provides future researchers with a model to conduct a cost-effectiveness analysis that considers the effects of exenatide on the prevention of PD. Third, several sensitivity analyses were performed to test the robustness of the results. Our findings supported the view that add-on exenatide is very cost-effective, even though the costs were thought to be relatively small compared to Western countries. For instance, the costs of PD were approximately US$10,146 to 23,101 in the US, whereas the costs in Taiwan were only half to one-fifth of the costs in the US (NT$141052.6) [82]. If the cost of DM reduction by exenatide is considered, the cost-effectiveness will be more prominent.

Our study has some limitations. First, the sample sizes in our PD cohort were small, and we could not obtain patients with H-Y stage 1 who were PD only; therefore, we replaced it with that in PD with DM. In addition, the relatively scarce information on H-Y stage 4^+^ may provide unrealistic medical utilisation. However, the trends in costs are in line with those in the literature and seem to be reasonable in late-stage PD patients.

The transition rate estimated from our PD cohort may inevitably include patients with DM. Nonetheless, our rates were still the lowest among other studies [53,54,81]. The effects of DM may have been diluted in this cohort. One explanation for this may be that early-stage PD was found by screening, in which accelerated progression brought about by DM was not yet significant.

Second, we did not consider DM development after PD due to an earlier onset age of DM compared to PD. According to these studies, PD alone may affect glycaemia and insulin tolerance. More evidence is needed to confirm the causal relationship between these two diseases. We chose a hypothesis that has been confirmed in many studies that preceding type 2 DM is a risk factor for PD. We did not consider the severity of DM due to the lack of published literature on this topic, and attainable information was insufficient. Our model could be modified if future studies unveiled a more underlying relationship between PD and DM.

Third, we could not directly obtain the costs of DM treated with exenatide because of the lack of local trials in Taiwan. The costs retrieved from the literature in Taiwan may include insulin. However, the results are cost effective. Retrospective or prospective studies are required to evaluate the efficacy and effectiveness of exenatide. Nowadays, a relatively higher price and non-oral type of exenatide have led to undesirability among patients with DM. Patients were started on GLP-1 agonists until the disease progressed to a more severe status in which insulin was needed. Thus, future studies using the NHIRD should be interpreted cautiously.

Fourth, the increase in utilities by exenatide came from a trial in which patients were not well controlled for HbA1c. This might not indicate the utilities that benefit from exenatide when used in the early stage. However, it remained cost-effective when we input the lowest utilities brought by exenatide in the sensitivity analysis.

Fifth, medication adherence was not considered in the model. It is unclear whether the impact of non-compliance would lead to PD and DM progression. In addition, multiple combinations of conventional pharmacotherapy made it difficult to evaluate non-adherence to specific drugs. Our model demonstrated perfect compliance, similar to that in RCT. In addition, insufficient evidence of the age-dependent efficacy of exenatide hinders us from a delicate model.

Lastly, it remains unclear whether the results can be extrapolated to countries other than Taiwan. Nonetheless, this model provides a reference for other countries as well. Finally, our data on cost were based on the NHRID between 2000 and 2009, when the data on PD were collected. This may also affect generalisation.

Add-on exenatide was demonstrated to be very cost-effective in PD combined with DM, which is based on a population-based viewpoint. Considering that DM may be a risk factor for neurodegenerative diseases, exenatide provides both clinical benefits and cost-effectiveness when considering both PD and DM.
